# Transformation of intimacy and its impact in developing countries

**DOI:** 10.1186/s40504-017-0056-8

**Published:** 2017-08-01

**Authors:** MD. Muniruzzaman

**Affiliations:** Department of Sociology and Anthropology, Green University of Bangladesh, Begum Rokeya Sarani, Dhaka, Bangladesh

**Keywords:** Intimacy, Relationship, Transformation, Sexuality, Reproduction, Erosion of values

## Abstract

Nowadays intimacy or intimate relationship is very familiar and widely used term all over the world. The term ‘Intimacy’ generally denotes a close interpersonal relationship or feeling of being in a close personal association and belonging together from both physical and mental point of view. It also denotes very close and effective connection with one another which may exist for whole life or may not. This article has been prepared on the basis of secondary sources and it tries to explore how this intimacy or intimate relationship has been gradually transforming from pre-modern society to modern society and from modern society to post-modern society for over the eras. This article also tries to explore the impact of transformed intimacy or intimate relationship, especially in the developing countries, like Bangladesh. Intimate relationship plays very significant role in the overall life style of any human being. This relationship includes feelings of liking, romance, sexuality or sexual relationship, emotional or personal support between mates. But the role of sexuality or sexual relationship is gradually increasing in intimacy, not only in the western countries but also in the developing countries. Nowadays people are involved with many kinds of premarital and extramarital relationships and they try to avoid the risk of reproduction. This tendency creates many problems in the developing countries, as most of the people of such developing countries are poor and illiterate. They are not aware about the dangerous impact of unsafe physical or sexual relationship. So the people of developing countries like Bangladesh are very vulnerable in the aspect of erosion of values and spreading different types of sexually transmitted diseases.

## Introduction

The concept of transformation of intimacy has been explained by Anthony Giddens^1^ in his famous book *‘The Transformation of Intimacy: Sexuality, Love, and Eroticism in Modern Societies’* that was published in 1992. He explained that how intimacy or the system of intimate relationships is gradually changing from one social stage to another. Intimate relationship generally denotes an interpersonal relationship particularly between male and female that involves physical, psychological and emotional intimacy. Giddens tries to explore and explain all these aspects of intimacy in his book.

In human relationships, the meaning and level of intimacy varies within and between relationships. In anthropological research, intimacy is considered the product of a successful seduction,^2^ a process of rapport building^3^ that enables parties to confidently disclose previously hidden thoughts and feelings. Intimate conversations become the basis for “confidences” (secret knowledge) that bind people together (Moore, 1985). To sustain intimacy for any length of time requires well-developed emotional and interpersonal awareness. Intimacy requires an ability to be both separate and together participants in an intimate relationship (Aronson, 2003).

Scholars classified intimacy in four types: **Physical**, **emotional**, **cognitive** and **experiential**. Physical intimacy is sensual proximity or touching, examples include being inside someone’s personal space, holding hands, hugging, kissing, petting and other sexual activity. Emotional intimacy, particularly in sexual relationships, typically develops after a certain level of trust has been reached and personal bonds have been established. The emotional connection of “falling in love”, however, has both a biochemical dimension, driven through reactions in the body stimulated by sexual attraction (Lowndes, 1996) and a social dimension driven by “talk” that follows from regular physical closeness or sexual union (Giddens, 1990). Cognitive or intellectual intimacy takes place when two people exchange thoughts, share ideas and enjoy similarities and differences between their opinions. If they can do this in an open and comfortable way, they can become quite intimate in an intellectual area. Experiential intimacy is when two people get together to actively involve themselves with each other, probably saying very little to each other, not sharing any thoughts or many feelings, but being involved in mutual activities with one another. Imagine observing two house painters whose brushstrokes seemed to be playing out a duet on the side of the house. They may be shocked to think that they were engaged in an intimate activity with each other, however from an experiential point of view, they would be very intimately involved (Healthy Place, 2008).

But all these types of intimacy or intimate relationships are not equally existed in all stages of our social system. These are gradually changed from one era to another. The intimacy or intimate relationships of pre-modernity, modernity and late-modernity (Giddens used the term late-modernity instead of post-modernity) are quite different. These changes of intimacy from one social stage to another may have both positive and negative impacts.

### Aims and objectives

The purpose of this article is to present a detailed description about intimacy or intimate relationship and its transformation from one social stage to another. The system, process and trends of intimacy are gradually transforming. But this transformation varies from one country to another; one society to another; and one era to another. To explore the variation of this transformation or changes of intimate relationships is another significant objective of this article. Like other countries of the world, the pattern of intimacy or intimate relationships is also transforming or changing in the developing countries like Bangladesh, India and Pakistan. This transformation of intimacy, especially in the developing countries has multidimensional impact. This paper also tries to explore such impact of transformation of intimacy in such kinds of developing countries.^4^

## Concept of transformation of intimacy

**Fig. 1:**
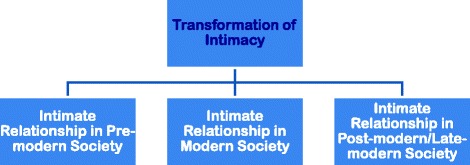
Transformation of Intimacy

Transformation of intimacy generally denotes how intimate relationships are transformed from pre-modernity to modernity and from modernity to post-modernity or late-modernity. The lifestyle and livelihood pattern, nature, attitude, behavior, emotion and sexuality of the people of pre-modern society are gradually changed in modern society. And all of these of modern society are also gradually changed in post-modern or late-modern society (Fig. 1).

Intimate relationship has over the last four or five decades evolved so far from its long-established ways—mutating in diverse directions—that its very nature and structuring, once a largely unquestioned given, is clearly up for some deep questioning and reformulating (Augustus, 2012) Changes of human intimacy or changes of all the aspects of intimate relationship which human being possess, lead to changes the overall social system. So with the transformation of intimacy from pre-modern society to modern society and from modern society to post-modern or late-modern society, the overall social system in these three phases is also transformed or changed.

### Intimate relationship in pre-modern society

Human history can be divided into three phases: pre-modern, modern and post modern. There is no definite beginning or end to each of these phases; rather they merge into one another, as not all societies moved forward at the same time. Although most industrialized countries are now considered post modern, a large proportion of the Third World^5^ remains modern or in some cases pre- modern. Pre- Modern is the period in society which came prior to Modernity, which began in Europe after the introduction of Industrial society and large scale production (UKessays, 2015). The Industrial Revolution, that started in Europe during the mid-1700s, is often the dividing line between pre-modern and modern societies; however, that’s not to say that today there still aren’t any semi-pre-modern societies left. They are just less likely to be found in an industrialized country like the United States, but they do still exist in some form here in our country. Also, if you were to travel to a place like Pakistan or Ghana, you would probably still find many pre-modern societies (Study.com).

In pre-modern society love, relationship and intimacy were not so popular like modern and post-modern societies. At that time ‘love’ generally means ‘love’ after marriage and partners were chosen by the parents especially in the developing countries. Because in pre-modern society marriages were conducted at very early ages and people had very less opportunity to fall in love before marriage. It has been known for a long time that the age of marriage, particularly for females, has been quite early in the Indo-Pakistan subcontinent (Ahmed, 1969). There are strong indications that the age of marriage for both males and females has been rising during the last half-century (Agarwala, 1962). In the pre-modernity, societies were agriculture based and developing countries were far behind from the advancement of science and technology. So, ‘love’ or intimate relationship, before marriage, was hardly seen here. Sometimes some love stories were found; and most of them were Persian or Arabian, but these also had a great impact among the people of Indian sub-continent. Let’s try to discuss two famous love stories of pre-modern society like that.

#### Love story of Laila and Majnu

**Fig. 2:**
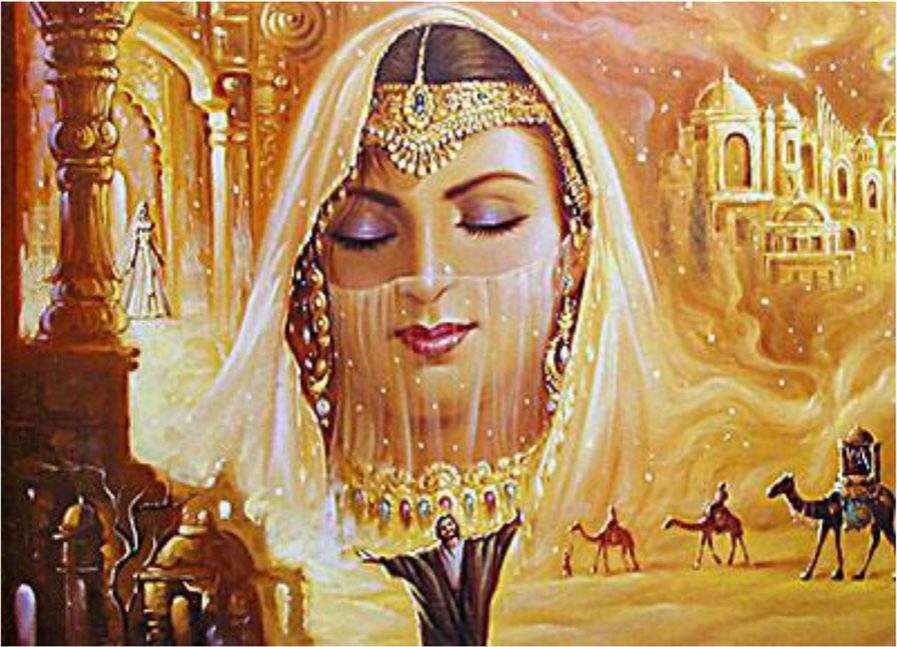
Tragic love story of Laila and Majnu

The love story of Laila and Majnu is a very famous one and is no less than a legend. They were two in flesh, but one in spirit. It is based on the real story of a young man called Qays ibn al-Mullawah from the northern Arabian Peninsula, in the Umayyad era during the seventh century. Laila was a beautiful girl born in a rich family. Being no less than a princess, she was expected to marry a wealthy boy and live in grandeur and splendor. But love is born from the heart; it knows no rules (Fig. 2).

Laila fell in love with Qays and he too loved her dearly. Qays was a poet and belonged to the same tribe as Laila. He composed splendid love poems and dedicated them to his lady-love, telling in them his love for her and mentioning her name often. Qays’ friends knew about his affair with Laila and they often teased and made fun of his love. But such taunts had no effect on Qays. He was deeply in love with Laila and it was her thoughts alone that possessed his mind for all time. It had been for quite some time that Qays toyed with the idea of seeking Laila’s hand in marriage from her parents. One day, he went up to them and put the big question before them. But Qays was a poor lad. And when he asked for Laila’s hand in marriage, her father promptly refused him as he didn’t want her daughter to marry below her status. It would mean a scandal for Laila according to Arab traditions. As fate would have it, the two lovers were banished from seeing each other. Soon after, Laila’s parents married her off to a wealthy man and she went on to live in a big mansion.

When Qays heard of her marriage he was heartbroken. He fled the tribe camp and wandered in the surrounding desert. His family eventually gave up on his return and left food for him in the wilderness. He could sometimes be seen reciting poetry to himself or writing Laila’s name in the sand with a stick. Day and night, he pined for her. Laila was no better. Seperated from Qays, she was shattered in mind, body and spirit. Not long afterwards, in 688 AD, she moved to Iraq with her husband, where she fell ill and died eventually. When Qays’ friends came to know about Laila’s death, they went looking for him all over to give him the news. But they could not find him. Not much later, their search for him came to an end. Qays was found dead in the wilderness near Laila’s grave. On a rock near the grave, he had carved three verses of poetry, which are the last three verses ascribed to him. Qays went mad for his love; for this reason he came to be called “Majnu”, or “Majnun Layla”, which means “Driven mad by Layla” (TheHolidaySpot, 2016 ).

#### The tragic love story of Shirin and Farhad

**Fig. 3:**
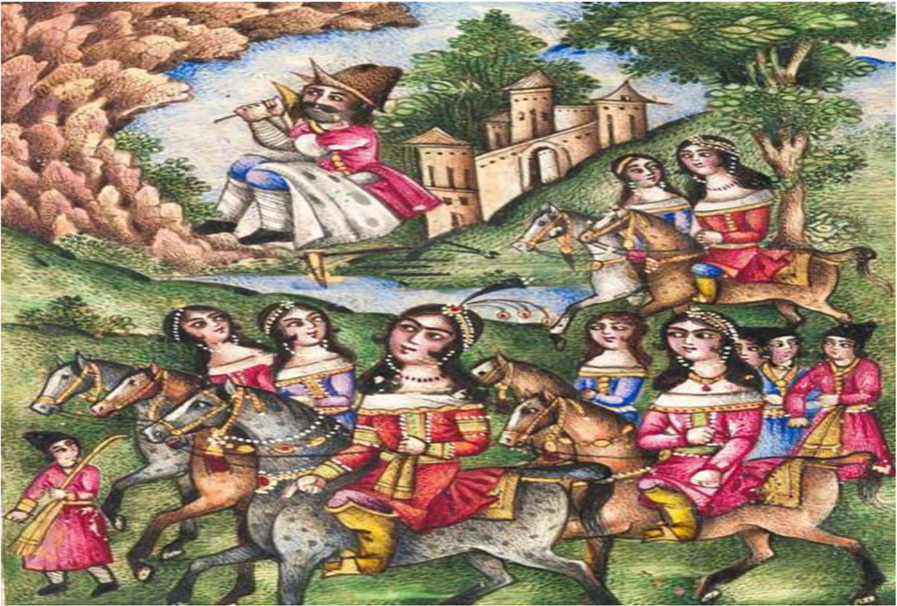
Tragic Love Story of Shirin and Farhad

The tragic love story of Shirin and Farhad is well known today, from Turkey to India and is especially popular in Iran. The encounter between Shirin and Farhad is part of a longer and much more tragic love story of Shirin and Khusrow. Farhad, the bearded man in the images, was a humble engineer, artist and craftsmen famed for his skillet carving rock, who served Shirin, the Queen of Armenia. Farhad fell in love with Shirin. In order to dissuade Farhad from his love for Shirin, Khusrow set him the impossible task of carving a tunnel through Mount Behistun. Before starting this arduous task, Farhad carved the likeness of Shirin into the rock face. It was that moment that is captured in these two miniature paintings. Farhad’s story does not end well. He is tricked by Khusrow into believing that Shirin has died, after which he kills himself using the tools that he had used to carve her very image into the rock (Mia.org) (Fig. 3).

Although these two tragic stories were Persian or Arabian, they had a great impact among the people of Indian sub-continent. Films were made in India about these two tragic love stories. For example, ‘Laila Majnu’ is a Hindi movie based on the legendary story of Layla and Majnun that was made in 1976. This film was directed by Harnam Singh Rawail and the stars Rishi Kapoor, Ranjeeta, Danny Denzongpa and Aruna Irani were the famous actors and actress. Similarly ‘Shirin and Farhad’ is another Hindi movie that was made in 1956. This film was directed by Aspi Azad and the stars Pradeep Kumar, Madhubala, Uma Dutt, Kamal, P Kailash, Ram Avtar, Shakuntala Paranjpye were the famous actors and actress. These two famous films regarding two tragic love stories had a great impact among the people of whole Indian subcontinent.

The Semitic romances of Shrin and Farhad, Laila and Majnun on their migration from Arabia and Persia, with the advent of the Muslims in India, have spread all over the Indian subcontinent. Besides being immensely popular in the respective places of their origin, they have crossed their native borders, have spread widely and have extended their influence all around. In course of time, they have become the vehicles of expression of personal emotion, empirical feelings, spiritual experiences and national sentiments of the people of those lands too (Shan, 2000).

These two tragic love stories are the examples of love and intimacy in pre-modern society when premarital love relationships were not as common as we see in the present era and the rate of success in love was also very low, particularly in Indian subcontinent.

### Intimate relationship in modern society

A modern society generally denotes a society that tries to continually move forward through its evolving ideas, norms, values, manners, practices and innovation. More specifically we can say that the idea of modern society is sort of social patterns that have been created as a result of industrialization. That means the era of modernity or modern society has been started from the very beginning of industrial revolution.^6^

Giddens concentrates on a contrast between traditional (pre-modern) culture and post-traditional (modern) culture. In traditional societies, individual actions need not be extensively thought about, because available choices are already determined (by the customs, traditions, etc.). In contrast, in post-traditional or modern society people (actors, agents) are much less concerned with the precedents set by earlier generations, and they have more choices, due to flexibility of law and public opinion. This however means that individual actions in modern society require more analysis and thought before they are taken. Society is more reflexive and aware; something Giddens is fascinated with, illustrating it with examples ranging from state governance to intimate relationships (Giddens, 2006). It is an interesting idea that romantic love is a product of modernity – or, at least, was accompanied with the process of modernization. Giddens assumes that romantic love began to make its presence felt from the late eighteenth century onward and that it is associated with ‘romance’, not only as a literature genre of novel but also as a form of storytelling in which self is narrated (Giddens, 1992).

Romantic relationships are voluntary and symmetrical, in contrast to the kinship or legal bonds that commonly circumscribe care giving relationships. Romantic relationships also involve dependency, which is reciprocal between the partners, unlike the more asymmetrical dependency of child on caregiver; and the reciprocal dependency of romantic partners is likely to be both greater and more extensive than the reliance of friends upon one another. Finally, romantic relationships are marked by an amalgam of love, passion, and actual or anticipated sexual activity (Andrew Collins and Alan Sroufe, 1999). Passion and sex, of course, have existed forever (Giddens, 1992). Romantic love, on the other hand, has not it represents the first phase of modern intimacy and was first recognized in the eighteenth century. This type of love is largely influenced by romantic novels, which increasingly became available to the masses by introducing ‘the idea of a narrative into an individual’s life’ (Giddens, 1992).

Romantic love is basically a type instantaneous attraction and it is monogamous. One partner is chosen for ever. That means romantic love is a love for ever. We can see the examples of these in many Indian, Pakistani and Bangladeshi films, plays, short stories, poems and novels. In Indian sub-continent, more specifically in India, Pakistan and Bangladesh, any kinds of premarital romantic relationships were so restricted from the late 18 century (When romantic love came into being according to Giddens) to late 20 century. At that time the success rate of these kinds of relationships were very low and the guardians or the parents did not allow these. But in the twenty-first century the success rate of premarital romantic relationships is relatively high and the guardians or the parents easily accept their children’s love relationships and allow them to marry.

### Intimate relationship in post modern or late modern society

The term ‘Postmodernism’ was first coined in the sociological and philosophical lexicon in 1979, with the publication of *The Postmodern condition: A report on Knowledge* by Jean-François Lyotard.^7^ The postmodern society or post modernity is the economic or cultural condition which is considered to exist especially in the western society after modernity or modern society. But some sociologists like Ulrich Beck, Zygmunt Bauman and Anthony Giddens repudiate the concept of post modernity and they argue that the process of modernization did not come to an end rather it continues into the contemporary era. So they are very much interested to use the term ‘late modernity’ or an advance stage of modernity instead of post modernity. Whether post modernity or late modernity, both indicate the present era which has been continuing since late 20 century. Revolutionary changes have been occurred in the aspect of intimacy or in the nature of human intimate relationships in post modern or late modern societies.

#### Confluent love

Confluent love is active and contingent. It jars with the forever, one and only qualities of romantic love. The emergence of confluent love goes some way towards explaining the rise of reparation and divorce. Romantic love meant that once people had married they were usually stuck with one another, no matter how the relationship developed. Now people have more choice: Whereas divorce was previously difficult or impossible to obtain, married people are now no longer bound to stay together if the relationship doesn’t work (Giddens, 2006). Unlike romantic love, confluent love is not necessarily monogamous, in the sense of sexual exclusiveness. What holds the pure relationship together is the acceptance on the part of each partner that, ‘until further notice’, that each gains sufficient benefit from the relation to make its continuance worthwhile. Sexual exclusiveness here has a role in the relationship to the degree to which the partners mutually deem it desirable or essential. In other words individuals remain in a relationship (albeit not necessarily exclusive) providing they are emotionally and sexually satisfied. Once this satisfaction is no longer present the individual chooses to end the relationship (Giddens, 1992).

#### Pure relationship

In describing late twentieth-century processes of social change, which involve a transformation in the nature of self-identity and intimacy, Giddens talks of the ascendancy of ‘confluent love’ and ‘the pure relationship’. Confluent love is contingent on lovers opening themselves out to each other. The ‘pure relationship’, like the ideal-typical dyad, has no overarching structure to sustain it. Rather, its key sustaining dynamics are mutual self-disclosure and appreciation of each other’s unique qualities (Jameison, 1999). Rather than basing relationships on romantic passion, people are increasingly pursuing the ideal of the pure relationship, in which couples remain because they choose to do so. Love is based upon emotional intimacy that generates trust. Love develops depending on how much each partner is prepared to reveal concerns and needs and to be vulnerable to the other. Each partner in the relationship constantly monitors their concerns to see if they are deriving sufficient satisfaction from the relationship for it to go on (Giddens, 2006).

There is a diversity of forms of pure relationship. Marriage can be one, though it is increasingly an expression of such a relationship once it already exists (as the number of couples cohabiting rises) rather than a way of achieving it. However, pure relationships are certainly not limited to marriage or indeed to heterosexual couples. In some forms, same-sex relationships, because of their open and negotiated status, come closer to the ideal of pure relationships than do heterosexual ones (Giddens, 2006).

#### Plastic sexuality

The concept of plastic sexuality is developed theoretically by Anthony Giddens (1992). “Plastic” refers to the malleability of erotic expression, in terms of both individual choice and frameworks of social norms. “Flexible sexuality” is argued to emerge in the context of the social changes in late modernity and post modernity. It stands in contrast to the features associated with modernist sexuality, conceptualized as fixed, by biology or by social norms. “Fixed sexuality” is associated with the binaries of modernity – either heterosexual or homosexual, either marital (legitimate) or extramarital (illegitimate), either committed or promiscuous, either normal (coital) or perverse (anal, autoerotic, sadomasochistic). For Giddens, plastic sexuality is the consequence of effective contraception, of the economic and social independence of women that also “liberated” men from the constraints of traditional gender expectations. Plastic sexuality is that which can be shaped according to individual erotic needs and wants. It can also serve as a marker of individual identity and/or as the means by which to make radical sexual demands. Thus, the consequence of disengaging sex from reproduction is to increase the emphasis on pleasure and decrease the emphasis on phallic sexuality. Giddens’s key claim for plastic sexuality is that it is “autonomous” sexuality (Blackwell Reference, 2016*).*

Giddens argues that the most recent phase of modernity has seen another transformation in the nature of intimate relationships. There has been the development of plastic sexuality. For people in these societies there is a much greater choice over when, how often and with whom they have sex than ever before. With plastic sexuality, sex can be untied from reproduction. This is partly due to improved methods of contraception, which have largely freed women from the fear of repetitive pregnancies and childbirths. However, it is not only technological developments that led to the emergence of plastic sexuality, but crucially the development of a sense of the self that could be actively chosen (Giddens, 2006).

#### Liquid love

The term ‘Liquid Love’ was explained by Polish sociologist Zygmunt Bauman^8^ in his book titled, *Liquid Love: On the Frailty of Human Bonds.* This book is about the central figure of our contemporary, liquid modern times -- the man or woman with no bonds, and particularly with none of the fixed or durable bonds that would allow the effort of self--definition and self--assertion to come to a rest. Having no permanent bonds, the denizen of our liquid modern society must tie whatever bonds they can to engage with others, using their own wits, skill and dedication. But none of these bonds are guaranteed to last. Moreover, they must be tied loosely so that they can be untied again (Bauman, 2003).

Bauman argues that in a world of rampant ‘individualization’ relationships are mixed blessings; they are filled with conflicting desires, which pull in different ways. On the one hand, there is the desire for freedom, for loose bonds that we can escape from if we so choose and for individualism. On the other hand, there is the desire for greater security that is gained by tightening the bonds between ourselves and our partners. As it is, Bauman argues, we swing back and forth between the two polarities of security and freedom. Often we run to experts – therapists or columnists, for example – for advice on how we can combine the two. The result is a society of ‘semi-detached couples’ in ‘top pocket relationships’. By the phrase ‘top pocket relationships’, Bauman means something that can be pulled out when needed, but pushed deep inside the pocket the moment they are not. Bauman even compares people’s attitudes to the relationship in ‘liquid modern’ society to the drink Ribena – in a concentrated from it is nauseating, and is best consumed diluted (Giddens, 2006).

‘Top -pocket’ relationships are characterized by their temporary and expendable nature. They are ‘sweet and short -lived’ and reflect a perfect embodiment of the instant and the disposable. Bauman stresses that one of the requirements for such a relationship involves the tacit agreement to avoid all encounters with love and desire: ‚ No love at first sight [...] No falling in love ... No sudden tide of emotions (2003: 21). The pivotal concept around which ‘top -pocket’ relationships are centered is convenience; after all this type of relationship relies on a lack of commitment to thrive. It strongly relates to the theory that the less one invests in a relationship, the less one loses when emotions falter or change completely (Bauman, 2003).

#### The normal chaos of love

In *The Normal Chaos of Love* (1995), Beck^9^ and Beck-Gernsheim^10^ examine the tumultuous nature of personal relationships, marriages and family patterns against the backdrop of a rapidly changing world. The traditions, rules and guidelines which used to govern personal relationships no longer apply, they argue, and individuals are now confronted with an endless series of choices as part of constructing, adjusting, improving or dissolving the union they form with others. The fact that marriages are now entered into voluntarily, rather than for economic purposes or at the urging of family, brings both freedoms and new strains (Giddens, 2006).

Men and women are increasingly becoming the authors of their own styles of life. The nature of love is changing fundamentally in conjunction with transformations in sexual life and family forms. Love, as Beck and Beck-Gernsheim argue, has become an empty category, which lovers themselves must fill in relation to their own biographies and emotional lives. The consequences of this situation are manifold. On the one hand, there stands the possibility of creating forms of democracy in personal life which parallel those achieved in the public sphere; on the other, there is the potentiality for chaos. Love, say the authors, becomes more important than ever before at the same time as it becomes more elusive. The struggle to harmonize family and career, love and marriage, ‘new’ motherhood and fatherhood has today replaced ‘class’ struggle. For better or for worse, individuals today who want to live together are becoming the legislators of their own ways of life, the judges of their own transgressions, the priests who absolve their owns sins and the therapists who loosen the bonds of their own past (Beck and Gernsheim, 1995).

## Impact of transformed intimacy in developing countries

Intimacy or the system of intimate relationships is not only transformed in developed or western countries but also in developing or 3rd world countries like Bangladesh, India and Pakistan. These three countries also have a great history of transformation. Before 1947, they were basically undivided Indo-Pakistan subcontinent. In 1947 this Indo-Pakistan subcontinent became independent from the British and two separate independent nations namely India and Pakistan have been emerged. After only 24 years, in 1971, the East part of Pakistan (present Bangladesh) became independent from the West with 9 months bloody and scarring Liberation war. As these three countries were undivided for a long time in history, their social settings resemble one another. Although some cultural differences are visible according to religions, castes and sects, the mainstream situation is almost similar. So the nature of intimacy of these countries is not so different. Confluent love, liquid love, top pocket relationship and plastic sexuality are also very common in these countries and these are gradually increasing. Flexibility of parents or guardians towards the premarital relationships of children, easy access of media, technology and internet, availability of different types of contraceptive methods and materials etc. are the reasons for the transformation of intimacy in Bangladesh and some other neighboring developing countries. However, let’s discuss the impact of transformation of intimacy in developing countries:

### Increasing divorce rate

Nowadays divorce rate is gradually increasing in South Asian developing countries. Recent data from a slum neighborhood in Dhaka suggest that about 15% of marriages there end in a divorce (Jesmin and Salway, 2000). At least in this urban slum setting, where marriages are more likely to be self-arranged rather than by parents or family, the weakening of family ties and social control, coupled with increased opportunities for re-partnering and economic independence of women may contribute to higher rates of divorce (Dommaraju and Jones, 2011).

Comparable data for India are lacking. There is, however, some data on divorce cases filed in family courts in cities, which shows an increase in divorce cases filed over the last two decades. In the family court in Mumbai (Bombay) the number of divorce cases filed increased from about 1800 in 1991 to about 2800 by 2001 (Singh and Sinha, 2005) and to about 4100 by 2007 (New York Times, 2008).

The divorce rate has been on the rise in Pakistan over the last decade. In Lahore city alone more than 100 divorces are registered in family courts in a day. The divorce rate is increasing not only in the upper class of society but also in lower and middle classes. From February 2005 to January 2008 approximately 75, 000 divorce cases had been registered. From February 2008 to May 2011, 1, 24,141 divorce cases were registered. Around 2, 59,064 separations have taken place in the provincial metropolis over the last decade. In 2010, 40,410 separation cases were registered in the city’s family courts and 13,500″ divorces have been filed so far in 2011 (Pakistan Today, 2011).

Lack of tolerance and trust, easy opportunity for remarriage, economic independence of women, easy opportunity for extramarital relationships etc. are the main causes of increasing divorce rate in these countries. Nowadays people don’t like to continue their matrimonial relationships after facing any problem.

However, for the majority of Asians, divorce and separation are still negative practices. Media reports say there is a dramatic increase in divorce rate in Bangladesh. Several reasons exist for this phenomenon, including extramarital relationship, economic growth, ‘marriage migrants’, familial influence, and the wider area of education. Women have become better educated, and begun enjoying the same opportunities as men, in terms of career development. In addition, a great number of abused wives decide to leave their husbands, feeling that they no longer need someone ‘stronger’ to watch over them. Thus the newer generation sees things differently. Dr. Mehtab Khanam of the Psychology department at Dhaka University comments, “Women are much more educated than before and are aware of their rights, so they don't tolerate anything which can affect their self-respect and dignity”. Bangladesh Mohila Parishad president, Advocate Elina Khan, says, “The divorce rate is increasing as a result of violence against women”. She added, “Women are now educated and self-reliant”. They are aware of their rights. If they find anything going wrong, such as extramarital relationship, they immediately seek relief. Sociologists and psychologists also express concern over the rising divorce rate. Professor Moniruzzaman of the Sociology Department at Dhaka University says it has also been found from newspaper reports that administration officials of Dhaka City Corporation’s 10 zonal offices said that 80% of the divorce petitions recorded with marriage registrars’ offices and courts in the city had been filed by women alleging extramarital affairs on the part of their husbands, torture by them and their intolerable behavior (Billah, 2013).

### No-fault divorce

No-fault divorce became an option in some states of U S A in the 1950s. Couples no longer needed to prove that one person was at fault. They could simply say that the marriage had broken down. By 1970, almost all states had laws allowing no-fault divorces. A long separation before the divorce used to be mandatory. Many states also passed laws that greatly decreased the separation time, making divorce easier and faster. These laws had a great effect on the divorce rate. From 1940 to 1965, the divorce rate remained near 10 divorces for every 1000 married women. By 1979, the rate had doubled (Attorneys). No-fault divorce is also gradually introducing in South East Asian developing countries like Indian subcontinent.

The Rajya Sabha, the upper house of the Indian parliament, approved an amendment to the Marriage Law in 2010 that would grant both men and woman the ability to file for divorce on the grounds of “irretrievable breakdown” of marriage. Both parties must live apart for 3 years before being able to file a petition for divorce on the grounds of “irretrievable breakdown” (jurist, 2015).

### Increasing extramarital relationship

An increasing number of couples are looking for love outside their homes, a study has found. Done by a city psychiatrist, the study reveals that 40% of the 500 people surveyed were involved in extramarital relationships. They cited reasons such as lack of love and attention from their spouse because of their hectic lifestyle and stress. Among married men in Bangladesh, those who reported having abused their wife physically, sexually or both in the previous year were more likely to report having premarital and extramarital sex partners than husbands who reported no such abuse, according to a nationally representative study conducted in 2004 (Silverman, 2007). As a Muslim majority country, extramarital relationship or extramarital sex is strongly prohibited in Bangladeshi society. Most of the Bangladeshi considers such kind of activities as Taboo. On top of that such kind of relationship is gradually increasing.

Extramarital affairs affect families in a multitude of ways. The man or woman who gets involved in extra marital affairs forgets that it might adversely affect the future of her/his family members. Like all other countries, extra marital affairs are the major factors leading to divorce in Bangladesh. This is due to the fact that extramarital affairs damage the trust in a relationship and trust is the predicator of all long-term relationships. There is tremendous damage done to the sense of trust between the partners that may never be recovered (Helal, 2013).

A research was conducted in Pakistan to explore the involvement of urban men in non marital or extramarital relationships. In order to determine the extent to which the study population engages in premarital or extramarital sexual activity, all respondents were asked to recall their non-marital sexual relation over their lifetime, within the last 12 and 3 months. Nearly one-third (29%) reported having had non-marital sex in their lifetime. Of thesemen16 percent reported premarital sex, while 11% reported engaging in both pre- and extramarital sex. Only 2 % reported exclusive extramarital sex. In total, 13% of men reported engaging in extramarital sexual activity (Mir et al., 2013).

### Step mother/father family and the challenges of children’s socialization

Young people in U.K. whose mother and father split up are also three times as likely to become aggressive or badly behaved, according to the comprehensive survey carried out by the Office for National Statistics. Living in a “reconstituted” family containing step-children or step-parents increased the risk of developing behavioral problems still further, it found. Children whose parents had split up over the 3 years were 4.53 times more likely to develop emotional problems than those whose mothers and fathers stayed together, and were 2.87 times more likely to show the onset of behavioral disorders (Beckford, 2008).

This problem is also increasing in India, Bangladesh and some other neighboring countries. Today step father or step mother and single parent families are increasing also in the developing countries of third world. In mid twentieth century, most single parent families came about because of the death of a spouse. In the 1970s and 1980s, most single parent families were the result of divorce (Kotwal and Prabhakar, 2009). Step parent family is very rare in Bangladesh. But now -a-days, as divorce rate is increasing, the step parent family is also increasing. Many studies in Bangladesh suggest that the children of step mother/father families get less opportunity to fulfill their basic needs. They have to face many challenges in their socialization process. Some of the children of step mother/father family become drug addict and juvenile delinquent.^11^

### High risk of spreading STDs and STIs

Although STDs and STIs are major health problem for both developing and developed countries, the developing countries like India, Bangladesh and Pakistan are more vulnerable in this regard. Because a large number of people of these countries are illiterate and they are not aware about HIV/AIDS, Syphilis, Gonorrhea, Hepatitis and other sexually transmitted diseases but their involvement in extramarital and premarital sexual intercourse is increasing day by day. Many poor women particularly slum dwellers in urban and semi urban areas of Bangladesh are working as floating CSWs (Commercial Sex Workers) but they have very poor knowledge about HIV/AIDS, Syphilis, Gonorrhea and other STDs.

Like other South Asian developing countries commercial sex works are very common in Bangladesh. Many married men have unprotected sex with sex workers and they also have unsafe or unprotected sex with their wives. As a result, not only these married men but also their wives are very much vulnerable for spreading HIV/AIDS and other STDs. Married women who only have sex with their husbands are at growing risk of HIV and sexually transmitted infections (STIs) due to the behavior of men who visit sex workers and sometimes engage in injecting practices (Habib, 2004). Besides, there are many brothels in urban and semi urban areas in Bangladesh where many CSWs are working to earn their livelihood. In Bangladesh there are about 14 acknowledged brothels. Among them seven are located in Dhaka division, six in Khulna division and only one, Patuakhali Brothel is in Barisal division. The brothels are generally located in or near the town, commercial area or river port (Alam, 2005). Rising trends in risk behavior was seen among adolescents in Bangladesh, including engaging in sex, suffering from STIs and having sex with commercial sex workers, this coupled with having limited knowledge regarding HIV/AIDS and limited access to RH services (Population Council and Frontiers, 2004).

The Indian epidemic is concentrated among vulnerable populations at high risk for HIV. The concentrated epidemics are driven by unprotected sex between sex workers and their clients and by injecting drug use with contaminated injecting equipment. Several of the most at risk groups have high and still rising HIV prevalence rates. According to India’s National AIDS Control Organization (NACO), the bulk of HIV infections in India occur during unprotected heterosexual intercourse. Consequently, and as the epidemic has matured, women account for a growing proportion of people living with HIV, especially in rural areas.

## Cause-effect relationship between the intimacy of post modernity or late modernity and some of their related impacts

Table 1 shows the intimacy of post modern society and some of their related impacts especially in developing countries. These impacts may be regarded as social problems as well and these social problems may have some other causes like poverty, rapid urbanization, migration etc. but the intimacy of post modernity also significantly responsible for these increasing social problems. Confluent love, liquid love, plastic sexuality etc. are basically polygamous type of intimacy in the sense of sexual exclusiveness, mostly seen in the post modern society, and these are the mentionable factors contributing to the rise of divorce rate, single parent/step parent family, premarital/extramarital sexual relationships, vulnerability of HIV/AIDS, STDs, STIs etc. particularly in developing countries.

**Table 1:** Cause-effect relationship between the intimacy of post modernity or late modernity and some of their related impacts

Intimacy of Post/Late Modernity	Mentionable Impact in Developing Countries
Confluent Love	• Increasing divorce rate • Increasing premarital relationships • Increasing extra marital relationship • Multiple simultaneous partners
Pure Relationship	• Increasing divorce rate • Increasing No-fault divorce • Increasing step mother/step father family • Challenges of children’s socialization
Plastic Sexuality	• Fulfilling physical needs without marriage • Fulfilling physical needs at anywhere, anytime • Multiple simultaneous partners • Postponement of marriage • High risk of spreading HIV/AIDS and other STDs
Liquid Love	• Tendency of changing partners • Increasing divorce rate • Increasing extra marital relationships • Increasing single parent family
Normal Chaos of Love	• Increasing divorce rate • Increasing no-fault divorce • Challenges of children’s socialization • Domestic violence

Divorce rates are not as high in Bangladesh as it is in developed countries. This may be because of the cultural and religious magnitude involved in the kinship and marriage systems in Bangladeshi society. But the past few decades have seen changes in marriage and family structures, and simultaneous developments in women’s legal and socio-economic status. The rate of divorce is increasing in Bangladesh (Parvez, 2011). Once, India used to have one of the lowest divorce rates in the world. Being a society largely based on a traditional value system, couples were both legally and socially dissuaded from seeking a divorce. However, socio-economic changes complemented by legal reforms in the last half a century, have enabled partners, especially women, to opt out of unequal and abusive marriages. The divorce cases of the last 5 years in Kerala give one an idea of where the state is headed for. The number was 8456 in 2005-‘06, 9775 in 2006-‘07, 9937 in 2007-‘08, 11,194 in 2008-‘09, 11,600 in 2009- ‘10 and 24,815 in 2010-‘11 (Premsingh and Rajan, 2014).

Nowadays tolerance and trust are gradually reducing in conjugal life. One partner always monitors the other which is labeled as ‘pure relationship’ by Giddens. This is one of the mentionable reasons for increasing extramarital relationships and divorce all over the world. More extramarital relationships, divorces and remarriages lead to more single parent and step parent families. The children of single parent and step parent families have to face many challenges in socialization process. In Bangladesh, after their parents’ separation, children face various problems due to economic insolvency. Because of severe poverty, most of them do not get educational facilities, health facilities and so on. They also go through an identity crisis. Besides, they cannot interact with their neighbors, peers, relatives and classmates. Most of them are always in frustration that leads to various deviant behaviors. They commit various crimes. Most of them are involved in drug addiction and smoking (Aktar, 2013).

Although premarital and extramarital sexual relationships are not as common in developing countries like India, Bangladesh and Pakistan as in Western countries, it is not as rare as perceived widely. A few studies in India mentioned that the adult students of schools and colleges have premarital sexual experiences. Neighbors, relatives, prostitutes, friends and fiancées have been mentioned as the partners of unmarried male students. There is an indication that the premarital sexual partner of a male student is often a married woman who may be a relative or neighbor. For example, one-half of all the first sexual partners of 72 college students in Hyderabad were married women older than themselves and a large majority of the partners are relatives (Nag, 1995). For these reasons they are very much vulnerable for spreading HIV/AIDS and other STDs. Globally, India ranks third, after South Africa and Nigeria, in the number of people living with HIV (Cohen, 2007).

Like other Asian countries Pakistan is also HIV epidemic, characterized by different risk factors. Formerly Pakistan was considered to be a low prevalence country, but now it is in the group of “Countries in Transition” with a concentrated epidemic among high risk groups, where the AIDS problem is increasing since last 5 years, according to the private newspaper The News and NACP NIH (Xinhuanet News, 2009). The number of infected persons might be running in millions if proper screening is carried out. The behaviors conducive to the spread of HIV infection to young people are curiosity about sex and drugs, negative peer pressure, and economic frustration in Pakistan (Abrar and Ghouri, 2010). Widespread poverty, significant power imbalances in men and women, labor migration, lack of any system to check the HIV positive reported persons, indiscriminate transfusion of unscreened blood, rising number of drug addicts and low condom use rates, are the serious risk factors that put the country in danger of facing a rapid spread of HIV (Human Development Report, Pakistan, 2009).

In spite of being a conservative Muslim country, Premarital and extramarital relationship is also very common in Pakistan. Both male and female are involved such kind of relationships. A study conducted by Malik et al.*,* (2014) at Dar-Ul-Aman^12^ on Pakistani married women reveals that married women run away with their boyfriends because of many socio-psychological, economic and religious factors. Enforced marriage, spousal abuse, age, impotency of husband, poverty of the husband, no understanding, verbal, psychological, physical violence, male domination and suppression, better economic well being of boy friend, emotional attachment with boy friend are the basic factors for involving in extra-marital relations and consequently their decision to get divorce from their husbands and opt for second marriage.

Conducting premarital and extramarital sexual intercourse is becoming very easy day by day in Bangladesh. While in Bangladesh the mainstream attitude towards sex and sexuality is still conservative, there is ample evidence that young people engage in premarital and extramarital sex, with the potential consequence of infection with HIV/STIs (NASP and MOHFW, 2010).

## Conclusion

From the above discussion we can conclude that as from the dawn of civilization society is dynamic and changeable, it is not unusual that intimacy or the system of human intimate relationships will be transformed from one social stage to another. But as social being, we have to consider the multidimensional impact of this transformation from various perspectives. Present trends of human intimacy like confluent love, liquid love, pure relationship, plastic sexuality etc. are more visible in western countries. But nowadays, these trends are gradually increasing also in the developing countries. As most of the people of developing countries are poor and illiterate, they cannot manage these types of transformations of intimacy in this post modern or late modern era like the people of developed western countries. Some poor and illiterate people like slum dwellers, especially in Bangladesh, by profession they are garment workers, rickshaw or van pullers, street hawkers, bus or truck drivers, boatmen, floating CSWs etc., are frequently involved in premarital and extra marital unsafe physical relationships without being aware of its dangerous impact. So, they are very much vulnerable for spreading HIV/AIDS and many other types of STDs. On the other hand confluent love or plastic sexuality or engaging in love relationship, sometimes physical relationships with many partners at a time is considerably seen among the young generations like school, college and university going boys and girls. These are creating many social problems like eve teasing, suicide, teen pregnancy etc. So, finally we can say that intimacy or the system of human intimate relationships may be changed or transformed, but we should not ignore the negative impact of it and self control and self consciousness are really very necessary in this aspect.

## Abbreviations

AIDS: Acquired immune deficiency syndrome
CSW: Commercial sex worker
HIV: Human immunodeficiency virus
NACO: National AIDS Control Organization
NACP: National AIDS control program
NIH: National Institutes of Health
RH: Reproductive health
STD: Sexually transmitted diseases
STI: Sexually transmitted infections
UNGASS: United Nations General Assembly Special Session

## Footnotes

1
**Anthony Giddens,** (born 18 January 1938) is a British sociologist who is known for his theory of structuration and his holistic view of modern societies. He is considered to be one of the most prominent modern sociologists, the author of at least 34 books, published in at least 29 languages, issuing on average more than one book every year. In 2007, Giddens was listed as the fifth most-referenced author of books in the humanities. (wikipedia).

2 Seduction means something that interests and attracts people or the act of persuading someone to have sex with him/her. (merriam-webster).

3 Rapport is getting on well with another person, or group of people, by having things in common, this makes the communication process easier and usually more effective (skillsyouneed).

4 A developing country is a nation or sovereign state with less developed industrial base and a low Human Development Index (HDI) relative to other countries (O’Sullivan and Sheffrin, 2003).

5 The economically underdeveloped countries of Asia, Africa, Oceania, and Latin America, considered as an entity with common characteristics, such as poverty, high birthrates, and economic dependence on the advanced countries. The French demographer Alfred Sauvy coined the expression (“tiers monde” in French) in 1952 by analogy with the “third estate,” the commoners of France before and during the French Revolution-as opposed to priests and nobles, comprising the first and second estates respectively. Like the third estate, wrote Sauvy, the third world is nothing, and it “wants to be something.” The term therefore implies that the third world is exploited, much as the third estate was exploited, and that, like the third estate its destiny is a revolutionary one. It conveys as well a second idea, also discussed by Sauvy, that of non-alignment, for the third world belongs neither to the industrialized capitalist world nor to the industrialized Communist bloc. The expression third world was used at the 1955 conference of Afro-Asian countries held in Bandung, Indonesia. In 1956 a group of social scientists associated with Sauvy’s National Institute of Demographic Studies, in Paris, published a book called Le Tiers-Monde. Three years later, the French economist Francois Perroux launched a new journal, on problems of underdevelopment, with the same title. By the end of the 1950’s the term was frequently employed in the French media to refer to the underdeveloped countries of Asia, Africa, Oceania, and Latin America (thirdworldtraveler).

6 The **Industrial Revolution** was the transition to new manufacturing processes in the period from about 1760 to sometime between 1820 and 1840. This transition included going from hand production methods to machines, new chemical manufacturing and iron production processes, improved efficiency of water power, the increasing use of steam power, the development of machine tools and the rise of the factory system. Textiles were the dominant industry of the Industrial Revolution in terms of employment, value of output and capital invested; the textile industry was also the first to use modern production methods (wikipedia.)

7 Jean Francois Lyotard (1924–1998) was a French post-structuralist philosopher, best known for his highly influential formulation of postmodernism in *The Postmodern Condition*. Despite its popularity, however, this book is in fact one of his more minor works. Lyotard’s writings cover a large range of topics in philosophy, politics, and aesthetics, and experiment with a wide variety of styles iep.utm.edu).

8
**Zygmunt Bauman** (born 19 November 1925) is a Polish sociologist. He has resided in England since 1971 after being driven out of Poland by an anti-semitic campaign engineered by the communist government of the Polish People’s Republic. Professor Emeritus of Sociology at the University of Leeds, Bauman is one of the world’s most eminent social theorists writing on issues as diverse as modernity and the Holocaust, postmodern consumerism and liquid modernity (wikipedia.org/wiki/Zygmunt_Bauman).

9
**Ulrich Beck** (May 15, 1944 – January 1, 2015) was a well known German sociologist, and one of the most cited social scientists in the world during his lifetime. His work focused on questions of uncontrollability, ignorance and uncertainty in the modern age, and he coined the terms “risk society” and “second modernity” or “reflexive modernization” (wikipedia.org/wiki/Ulrich_Beck).

10
**Elisabeth Beck-Gernsheim** (born 14 October 1946), is a German sociologist, psychologist, and philosopher. She holds a professorship at the University of Erlangen-Nuremberg. After studying sociology, psychology and philosophy in Munich, she obtained her PhD in 1973. After several fellowships, she graduated from Munich University in 1987.Her main research interest is in social changes and the changing situation of the institution of the family. She is the widow of the late sociologist, Ulrich Beck (wikipedia.org/wiki/Elisabeth_Beck-Gernsheim).

11 Juvenile delinquents are minors, usually defined as being between the ages of 10 and 18, who have committed some act that violates the law. These acts aren’t called “crimes” as they would be for adults. Rather, crimes committed by minors are called “delinquent acts.” Instead of a trial, the juvenile has an “adjudication,” after which she receives a “disposition” and a sentence (criminal.findlaw).

12 Dar-ul-Aman is basically an institution that protects the women who are deprived of family support due to some reasons. Its purpose is to rehabilitate needy women either by arranging, settlement or marriages after the decision of the cases or to help in their certain life affairs (Malik et al., 2014).
